# Urinary protein biomarkers based on LC–MS/MS analysis to discriminate vascular dementia from Alzheimer’s disease in Han Chinese population

**DOI:** 10.3389/fnagi.2023.1070854

**Published:** 2023-01-25

**Authors:** Ruijuan Chen, Yuanjing Yi, Wenbiao Xiao, Bowen Zhong, Le Zhang, Yi Zeng

**Affiliations:** ^1^Department of Geriatrics, Second Xiangya Hospital, Central South University, Changsha, Hunan, China; ^2^Department of Emergency, Central South University Xiangya School of Medicine Affiliated Haikou Hospital, Haikou, Hainan, China; ^3^State Key Laboratory of Proteomics, Beijing Proteome Research Center, National Center for Protein Sciences (Beijing), Beijing Institute of Lifeomics, Beijing, China; ^4^Department of Neurology, Xiangya Hospital, Central South University, Changsha, Hunan, China

**Keywords:** vascular dementia, Alzheimer’s disease, biomarkers, urine, proteomics

## Abstract

**Objective:**

This study aimed to identify the potential urine biomarkers of vascular dementia (VD) and unravel the disease-associated mechanisms by applying Liquid chromatography tandem-mass spectrometry (LC–MS/MS).

**Methods:**

LC–MS/MS proteomic analysis was applied to urine samples from 3 groups, including 14 patients with VD, 9 patients with AD, and 21 normal controls (NC). By searching the MS data by Proteome Discoverer software, analyzing the protein abundances qualitatively and quantitatively, comparing between groups, combining bioinformatics analysis using Gene Ontology (GO) and pathway crosstalk analysis using Kyoto Encyclopedia of Genes and Genomes (KEGG), and literature searching, the differentially expressed proteins (DEPs) of VD can be comprehensively determined at last and were further quantified by receiver operating characteristic (ROC) curve methods.

**Results:**

The proteomic findings showed quantitative changes in patients with VD compared to patients with NC and AD groups; among 4,699 identified urine proteins, 939 and 1,147 proteins displayed quantitative changes unique to VD vs. NC and AD, respectively, including 484 overlapped common DEPs. Then, 10 unique proteins named in KEGG database (including PLOD3, SDCBP, SRC, GPRC5B, TSG101/STP22/VPS23, THY1/CD90, PLCD, CDH16, NARS/asnS, AGRN) were confirmed by a ROC curve method.

**Conclusion:**

Our results suggested that urine proteins enable detection of VD from AD and VC, which may provide an opportunity for intervention.

## 1. Introduction

Dementia is a clinical syndrome triggered by many differing pathologies, mainly including Alzheimer’s disease dementia (AD), vascular dementia (VD) and mixed AD/VD, dementia with Lewy bodies (DLB) and frontotemporal dementia (FTD) (Kokkinou et al., 2021). In 2030, it was estimated that there will be about 75.6 million people with dementia and the numbers are projected to rise to 131.5 million by 2050 (Ijaopo, 2017). Due to the incurable character of the disorder, most patients in aging often suffer various complications, high frequency of readmission, poor self-care ability, heavy psychological and physical burden, which may take up extensive social and economic resources. Therefore, the prevention of dementia relying on early diagnosis and distinction is a priority. Great effort has been made over the past decade to identify early and accurate disease-specific biomarkers as optimized tools for the differential diagnosis of dementia subtypes (Llorens et al., 2016; Maul et al., 2020). In 2020, Bai et al. demonstrated new potential proteins and molecular networks in brain tissue, cerebrospinal fluid during the progression of AD through proteomics based on mass spectrometry (MS) and multilayer omics methods, and further identified them in mouse models (Bai et al., 2020). However, to date, there are no valid options for the earliest possible and reliable diagnosis, but so far, these candidate biomarkers have only been established by AD, mostly requiring invasive and expensive procedures (Maul et al., 2020; Kokkinou et al., 2021). The clinical diagnosis of VD still depends on imaging criteria and there is no specific biochemical marker.

The Label-free proteomics quantitative techniques based on Liquid chromatography tandem-mass spectrometry (LC–MS/MS) are simple and practical, and play an important role in clinical research, biomarker discovery and personalized medicine recently (Krey et al., 2014; Zhao et al., 2020). Unprecedented proteomic coverage, such as in brain tissue, cerebrospinal fluid, and serum, has yielded new putative AD biomarkers (Bai et al., 2021). In addition, urine is an excellent biospecimen utilized as a desirable source of protein-biomarker discovery, because it can be collected recurrently by non-invasive techniques and reflect pathophysiologic state, especially in the early stages of disease (Barratt and Topham, 2007; Harpole et al., 2016). In addition, urine biomarkers could be used not only to diagnose but also to monitor the pathological changes of disease therapeutics (Zhang et al., 2018). The goals of this study, we sought to use LC–MS/MS label-free methods to characterize the urine proteome in VD, AD and normal controls (NC) subjects to identify disease-associated alterations in proteins and biological pathways. Furthermore, we aimed to identify urine proteins that could distinguish VD from both NC and AD by applying feature analysis to our urine proteomic profiles.

## 2. Materials and methods

### 2.1. Ethics approval

This study was approved by the Ethics Committee of the Second Xiangya Hospital of Central South University, and all participants provided written informed consent following the Declaration of Helsinki and the independent ethics committee or institutional review board.

### 2.2. Study design and population

**Figure 1:**
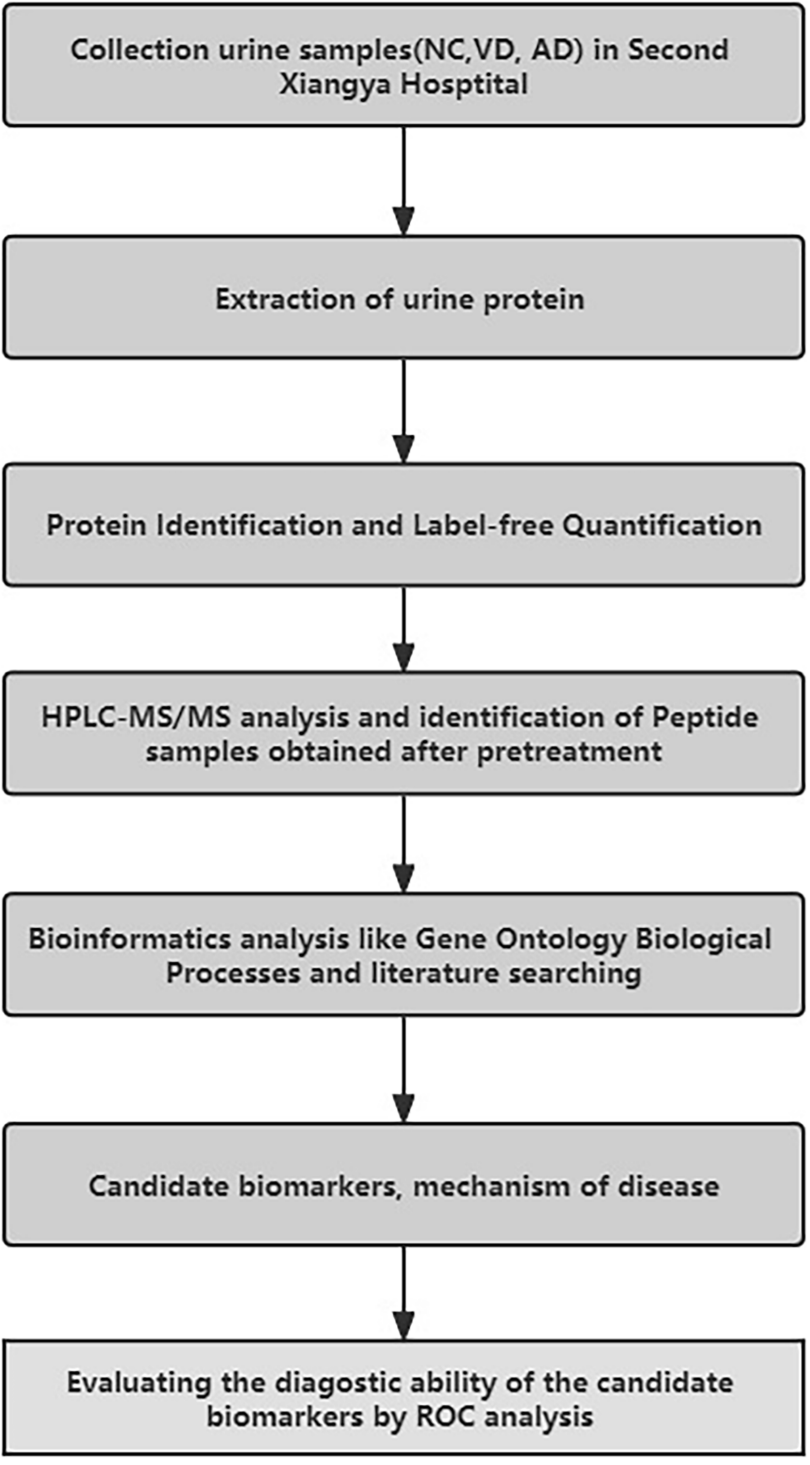
The work flow of the experimental procedures.

The workflow of this study is shown in Figure 1. In total, 44 subjects were recruited in the Second Xiangya Hospital of Central South University from 2016 to 2017, including 14 patients with VD, 9 patients with AD, 21 NC subjects matched with their age and sex from the Health Examination Center of the Second Xiangya Hospital during the same period, with no history of chronic disease.

### 2.3. Inclusion/exclusion criteria and clinical examination

All VD patients were diagnosed by neurologists according to the criteria defined by the National Institute of Neurological Disorders and Stroke-Association International pour la Recherche et l’Enseignement en Neurosciences (NINDS-AIREN) (Román et al., 1993; Pohjasvaara et al., 2000) and the central assessment of the neuroimaging criteria for VD. All patients with VD must have a history of stroke or transient ischemic attacks at least 3 months earlier attributed to large artery atherosclerosis, small artery occlusion, cardioembolism et al. And patients with lobar hemorrhages or space-occupying lesions were excluded. The evaluation of all subjects as cognitively normal was based on comprehensive neuropsychological testing (Almeida et al., 2015) by using the scales such as Mini-Mental Status Examination (MMSE), Clock Draw Test (CDT), Verbal Category Fluency Test (VCFT), Hamilton Depression Scale (HAMD), Hamilton Anxiety Scale (HAMA). Classification of AD was mainly based on the evidence of the neuropsychological manifestations and comprehensive neuropsychological testing, except for the evidence of amyloid beta (Aβ) accumulation on Positron emission tomography (PET) imaging due to expensive expenses, as defined by Current research diagnostic criteria from the National Institute on Aging and the Alzheimer’s Association (NIA-AA) (Albert et al., 2011; McKhann et al., 2011).

### 2.4. Urinary samples collection and processing

All the first morning urinary samples were collected in polypropylene tubes according to standardized local procedures and stored at –80°C after centrifugation as our prior study (Chen et al., 2021).

### 2.5. LC–MS/MS analysis

A total of 44 urinary proteins resuspended in the buffer solution were digested overnight with Trypsin (Promega, United States) at 37°C temperature, acidified with TFA, redissolved with acetonitrile (ACN, Sigma, United States), eluted using two solvent buffer (A: 99.9% water and 0.1% formic acid; B: 79.9% ACN, 20% water, and 0.1% formic acid), and analyzed by using a Q Exactive HF quadrupole-Orbitrap mass spectrometer (Thermo Fisher Scientific) coupled to UltiMate 3,000 HPLC and UHPLC Systems (Thermo Fisher Scientific). Differential proteins were identified and quantified against the complete human proteins in the Uniprot database (2020.07.02) using Proteome Discover 2.4 software (Thermo Fisher Scientific) with SEQUEST and Mascot search engine (version 2.3.01, Matrix Science, London, United Kingdom). Then bioinformatics and statistical analysis were essentially performed as described previously: (1) differentially abundant proteins from discovery proteomics were selected using t-test after log2 transformed ratio based on the following criteria: *p* < 0.05 and FC < 0.83 or > 1.20; (2) the demographic data were presented as mean ± SEM, and statistical analysis was performed by the two-tailed t-test and one-way ANOVA for the comparison between groups; *p* < 0.05 was statistically significant. All procedure has been reported in details in our prior study (Chen et al., 2021).

### 2.6. Evaluating the diagnostic ability of candidate markers

The ROC curve method was used to qualify the performance of single candidate markers screened by the proteomic analysis, between the VD group with other two groups (as a whole group; Abdi et al., 2006). Wilcoxon Rank-Sum test was used to determine the statistical significance of a single marker and evaluate the significance of the entire ROC curve.

## 3. Results

### 3.1. Baseline characteristics

Demographic and clinical characteristics of participants in this study were summarized in Table 1. There were no significant differences in age, years of education, between the VD, AD and NC groups. The majority of patients of the VD group had similar clinical profiles: the history of hypertension, stroke/transient ischemic attacks at least 3 months before and a high HIS score compared with other groups. Both VD and AD groups have a significantly lower score of MMSE, clock-drawing test (CDT), and verbal category fluency test (VCFT); and a higher score of HAMA. All assessments of MMSE, CDT, VCFT, HAMD, and HAMA of NC group were normal. Compared with the other two groups, AD group showed a slight increase in HAMD score.

**Table 1:** Demographic and clinical characteristics of subjects and numbers of sample for NC, VD, AD group.

	VD (*n* = 14)	AD (*n* = 9)	NC (*n* = 21)	*p* value
Sex(female/male)	5/9	5/4	7/14	–
History of hypertension (yes/no)	7/7	3/6	4/17	–
History stroke/transient ischemic attacks for at least 3 months (yes/no)	14/0	0/9	0/21	–
Age(year)	76.00 ± 8.03	79.89 ± 9.96	72.67 ± 8.99	0.13
Years of education	8.50 ± 2.11	7.00 ± 1.94	7.33 ± 1.27	0.07
HIS	11.85 ± 1.87	3.80 ± 1.17	2.44 ± 0.68	<0.0001
MMSE	15.92 ± 4.39	14.89 ± 4.04	29.4 ± 0.55	<0.0001
CDT	1.25 ± 0.97	1.22 ± 0.91	4.00 ± 0.00	<0.0001
VCFT	7.50 ± 3.03	9.33 ± 2.58	15.40 ± 0.89	<0.0001
HAMD	6.67 ± 1.61	9.56 ± 2.41	6.20 ± 1.30	<0.0001
HAMA	8.08 ± 3.63	7.33 ± 1.89	2.80 ± 1.48	<0.0001

CRE, serum creatinine; HIS, Hachinski score; MMSE, Mini-Mental Status Examination; CDT, Clock Draw Test; VCFT, Verbal Category Fluency Test; HAMD, Hamilton Depression Scale; HAMA, Hamilton Anxiety Scale.

#### 3.1.1. Proteomics findings

**Figure 2:**
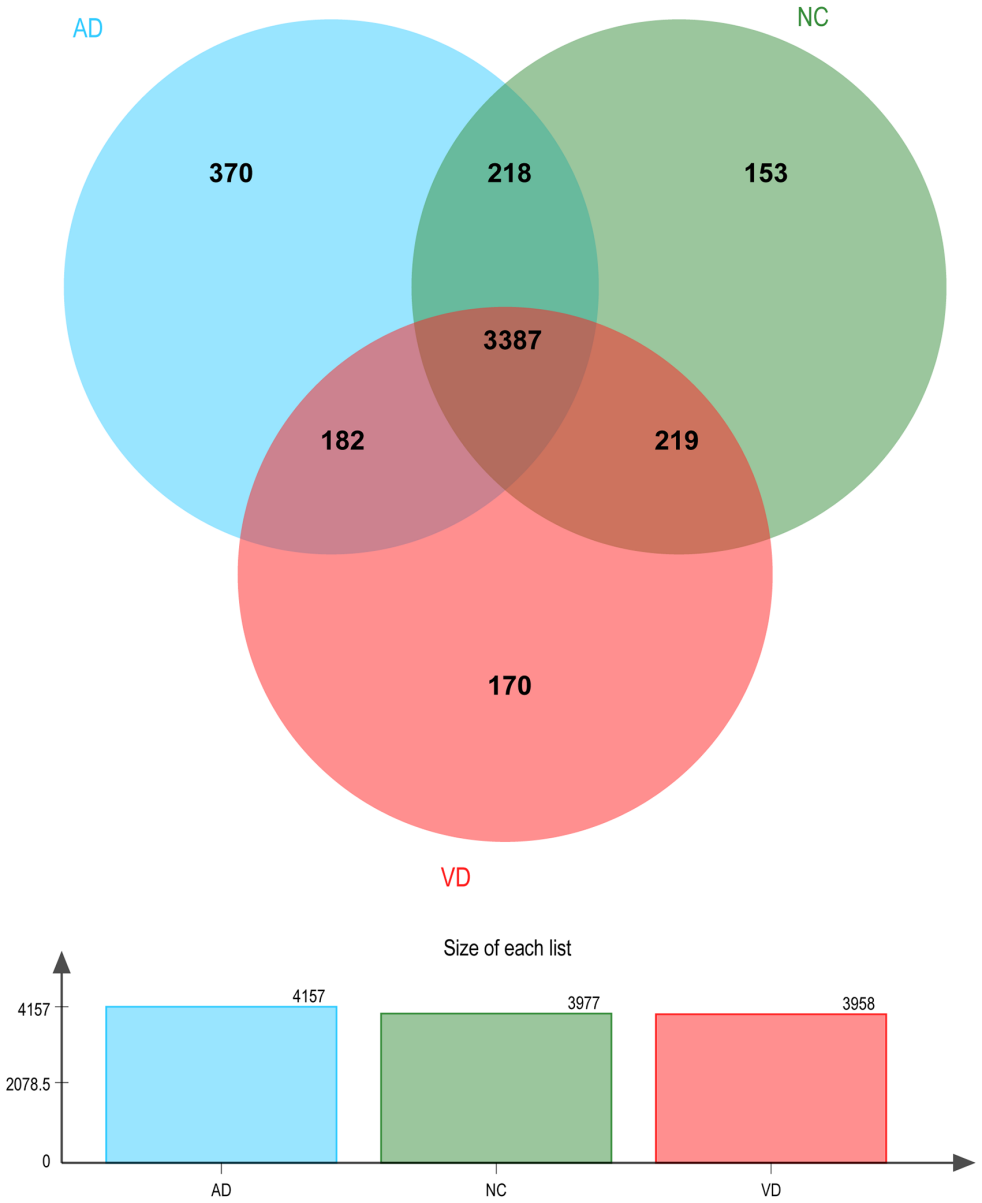
Venn diagram illustration of Urine proteins identified in VD, AD, and NC group, respectively. Representative Venn diagrams showing common proteins found in pooled urine-enriched fractions from VD patients (*n* = 14), AD patients (*n* = 9), and NC group (*n* = 21).

A total of 4,699 urinary proteins were successfully identified (Supplementary Table 1), out of which 3,958, 4,157, and 3,977 urinary proteins were identified in VD, AD, and NC groups, respectively. Three thousand three hundred eighty seven proteins were found to be common shared among the three groups (Figure 2), which demonstrated that they share the overlapping physiopathologic mechanism of protein molecules.

### 3.2. Differential proteins identified

#### 3.2.1. VD vs. NC

**Figure 3:**
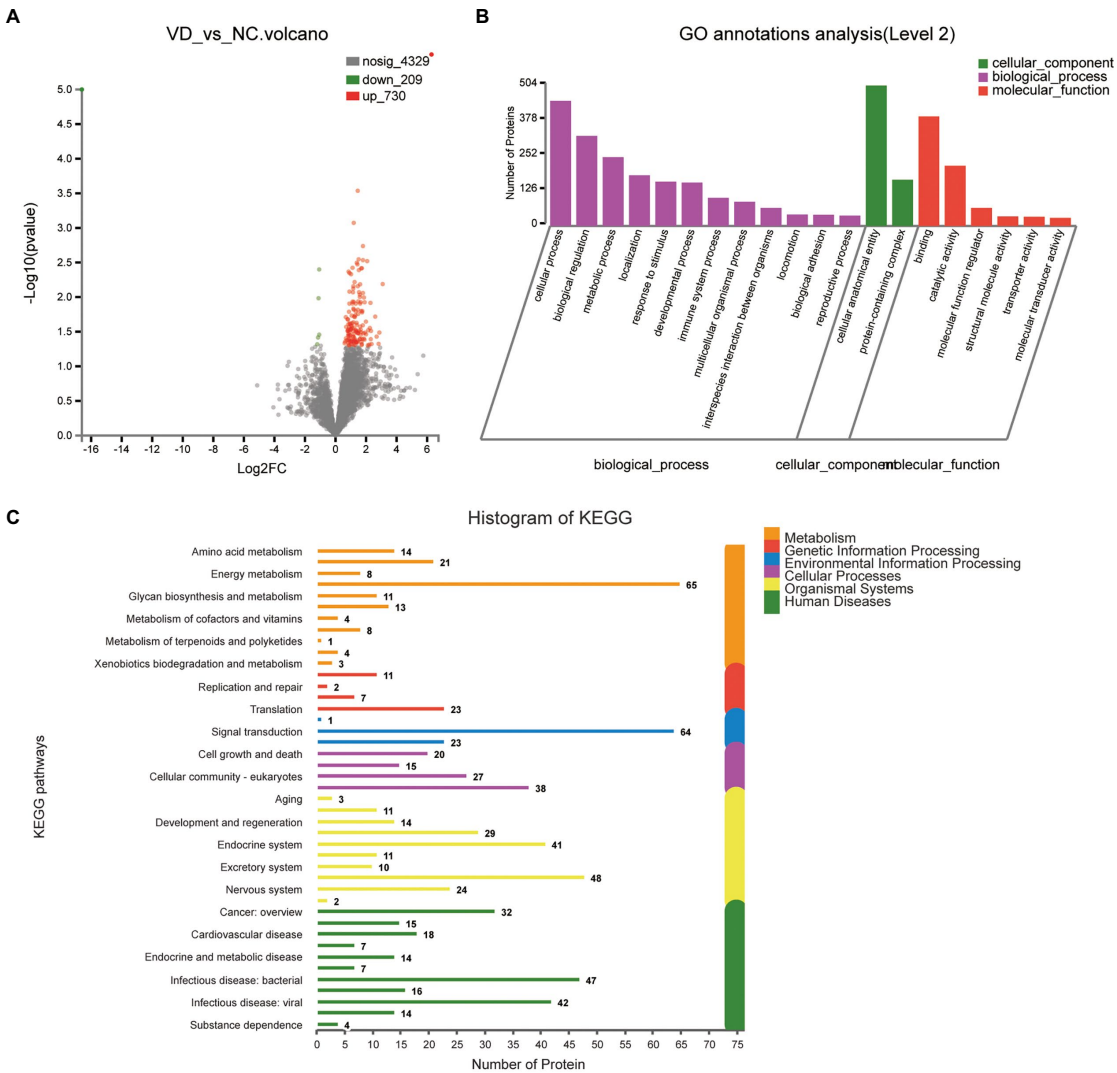
Volcano plots of quantified proteins and functional analysis of the differentially expressed proteins in the urine between VD and NC subjects (Notes same as figure 3-1). **(A)** Volcano plot of mean TMT log 2 ratio plotted against the minus log 10 (value of *p*) of the one-sample *T*-test for the respective quantified proteins. Each point in the figure represents a specific protein. The point on the left is the protein with down-regulated expression, and the point on the right is the protein with up-regulated expression. Green points represent down-regulated differential proteins and red points represent up-regulated differential proteins. **(B)** GO enrichment analysis of differential urine-excretory proteins. Purple bars denote the enriched biological processes, green bars are enriched cellular components, and red bars are enriched molecular functions. Protein numbers enriched in each category are presented with the bars. **(C)** The differentially expressed proteins are associated with diseases and signal transduction pathways. The number of proteins associated with each category is listed. The different color pathways represent the different categories to which they belong. According to the KEGG metabolic pathways involved in proteins, they can be divided into 7 categories: Metabolism (M), Genetic Information Processing (GIP), Environmental Information Processing (EIP), Cellular Processes (CP), Organismal Systems (OS), Human Diseases (HD), Drug Development (DD).

A total of 939 DEPs differentially identified by VD vs. NC, including 730 upregulated genes and 209 downregulated genes were encoded, were predicted to be urinary excretion in two groups (Figure 3A). Details of these DEPs were provided in Supplementary Table 2. GO enrichment analyses were further conducted by using Blast2GO as our prior study (Chen et al., 2021). The enriched GO terms (Level 2) include biological processes, cellular components, and molecular functions (Figure 3B). In cellular component, these proteins were mainly enriched in protein-containing complex (166 DEPs), cellular anatomical entity (504 DEPs). In biological processes, these proteins were mainly enriched in cellular process (449 DEPs), biological regulation (323 DEPs), metabolic process (247 DEPs), localization (182 DEPs), response to stimulus (159 DEPs), developmental process (156 DEPs), immune system process (101 DEPs), multicellular organismal process (87 DEPs), interspecies interaction between organisms (65 DEPs), locomotion (41 DEPs), biological adhesion (40 DEPs), reproductive process (37 DEPs) etc. In molecular function, these proteins were mainly enriched in binding (393 DEPs), catalytic activity (216 DEPs), molecular function regulator (65 DEPs). According to the KEGG metabolic pathway that proteins participate in, they can be divided into 7 categories: Metabolism (M), Genetic Information Processing (GIP), Environmental Information Processing (EIP), Organismal Systems (OS), Human Diseases (HD), Drug Development (DD), Organismal Processes (CP), Organismal Systems (OS), Human Diseases (HD), Drug Development (DD). In M pathways, 21, 14, 13, 11, 8 proteins were separately annotated for the Carbohydrate metabolism, Amino acid metabolism, Lipid metabolism, Glycan biosynthesis and metabolism Energy metabolism. According to the HD pathway analysis, 24 proteins were annotated for Nervous system and 14 proteins were annotated for neurodegenerative diseases (Figure 3C).

#### 3.2.2. VD vs. AD

**Figure 4:**
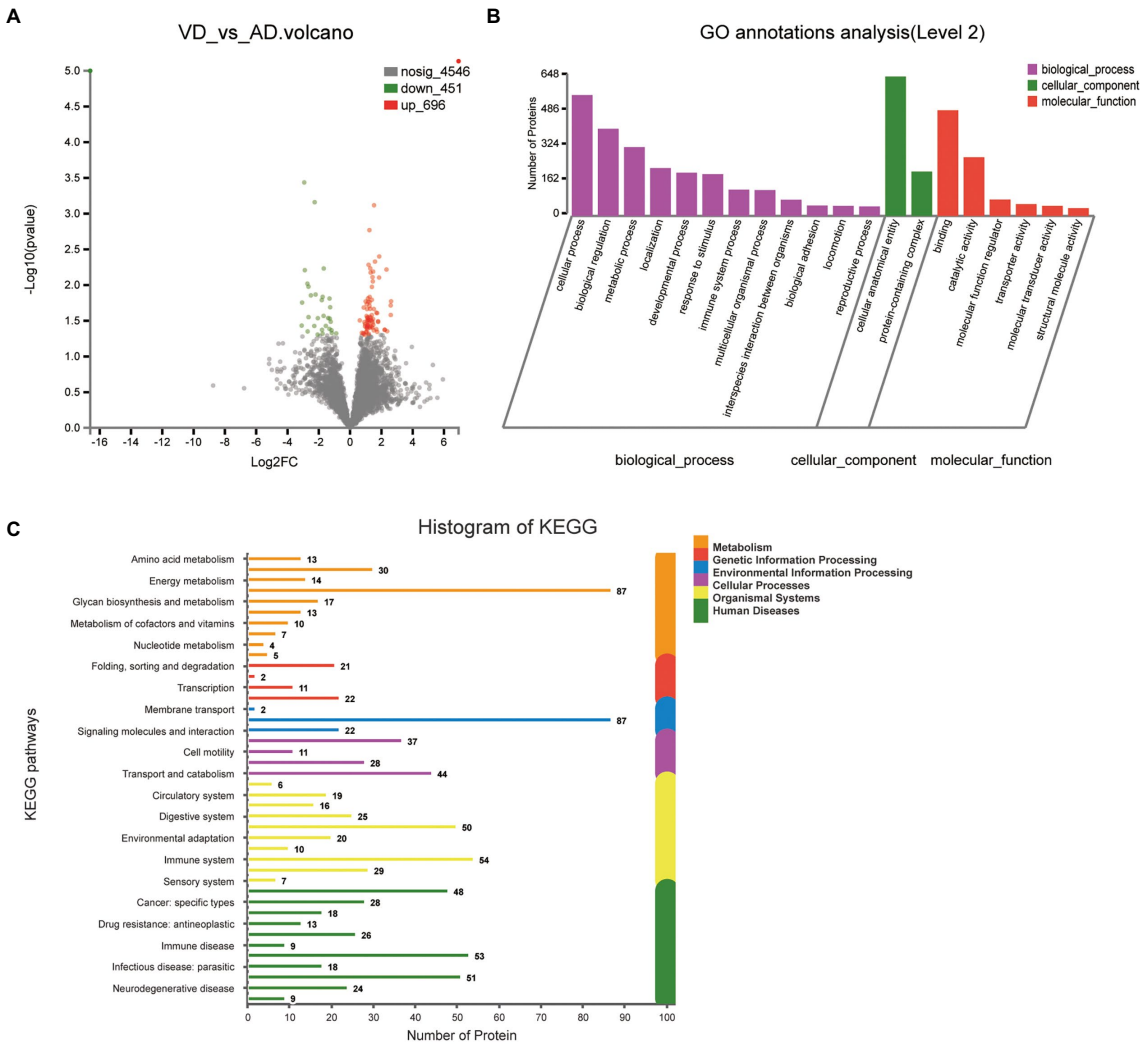
Volcano plots of quantified proteins and functional analysis of the differentially expressed proteins in the urine between VD and AD groups. **(A)** Venn chart of Urine-based Proteome analysis identified from VD vs AD. **(B)** GO enrichment analysis of differential urine-excretory proteins. Purple bars denote the enriched biological processes, green bars are enriched cellular components, and red bars are enriched molecular functions. Protein numbers enriched in each category are presented with the bars. **(C)** The differential expressed proteins are associated with diseases and signal transduction pathways. The number of proteins associated with each category is listed.

One thousand one hundred forty seven DEPs were identified by comparing VD and AD subjects, in which 696 proteins were upregulated and 451 proteins were downregulated (Figure 4A). Though the main biological processes enriched GO terms were similar to VD vs. NC, the number of DEPs annotated to each pathway varies (Figure 4B). Details of these DEPs were provided in Supplementary Table 3. In biological processes, these proteins were mainly enriched in cellular process (562 DEPs), biological regulation (405 DEPs), metabolic process (320 DEPs), localization (222 DEPs), developmental process (201 DEPs), and response to stimulus (194 DEPs). In molecular function, these proteins were mainly enriched in binding (491DEPs), catalytic activity (273 DEPs), molecular function regulator (76 DEPs). In cellular component, these proteins were mainly enriched in protein-containing complex (206 DEPs), cellular anatomical entity (648 DEPs). 48, 18, 13, 26, 9, 53 proteins were separately annotated for Cancer: overview, Cardiovascular disease, Drug resistance: antineoplastic, Endocrine and metabolic disease, Immune disease, Infectious disease: bacterial, and 29 proteins were annotated for Nervous system and 24 proteins were associated with neurodegenerative diseases by the KEGG metabolic pathway analysis (Figure 4C). Seven DEPs enriched in the pathway of neurodegenerative diseases overlapped with VD vs. NC.

#### 3.2.3. Common differential proteins of VD compared with NC, AD group separately

**Figure 5:**
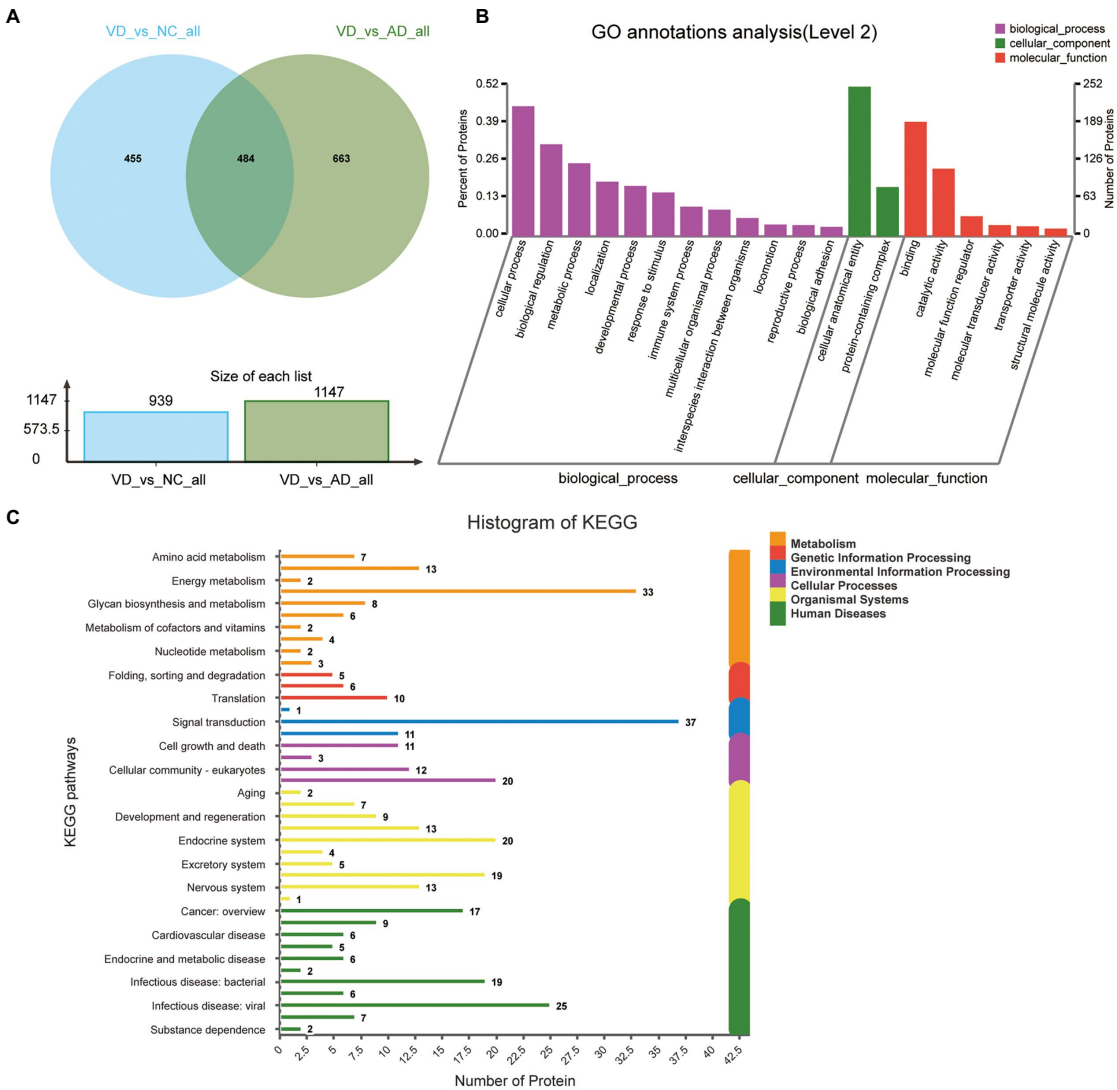
Volcano plots of quantified proteins and functional analysis of the differential expressed proteins in urine between VD and other two groups. **(A)** Venn chart of Urine-based Proteome analysis identified from VD vs. other two groups. **(B)** GO enrichment analysis of differential urine-excretory proteins. Purple bars denote the enriched biological processes, green bars are enriched cellular components, and red bars are enriched molecular functions. Protein numbers enriched in each category are presented with the bars. **(C)** The differential expressed proteins are associated with diseases and signal transduction pathways. The number of proteins associated with each category is listed.

**Figure 6:**
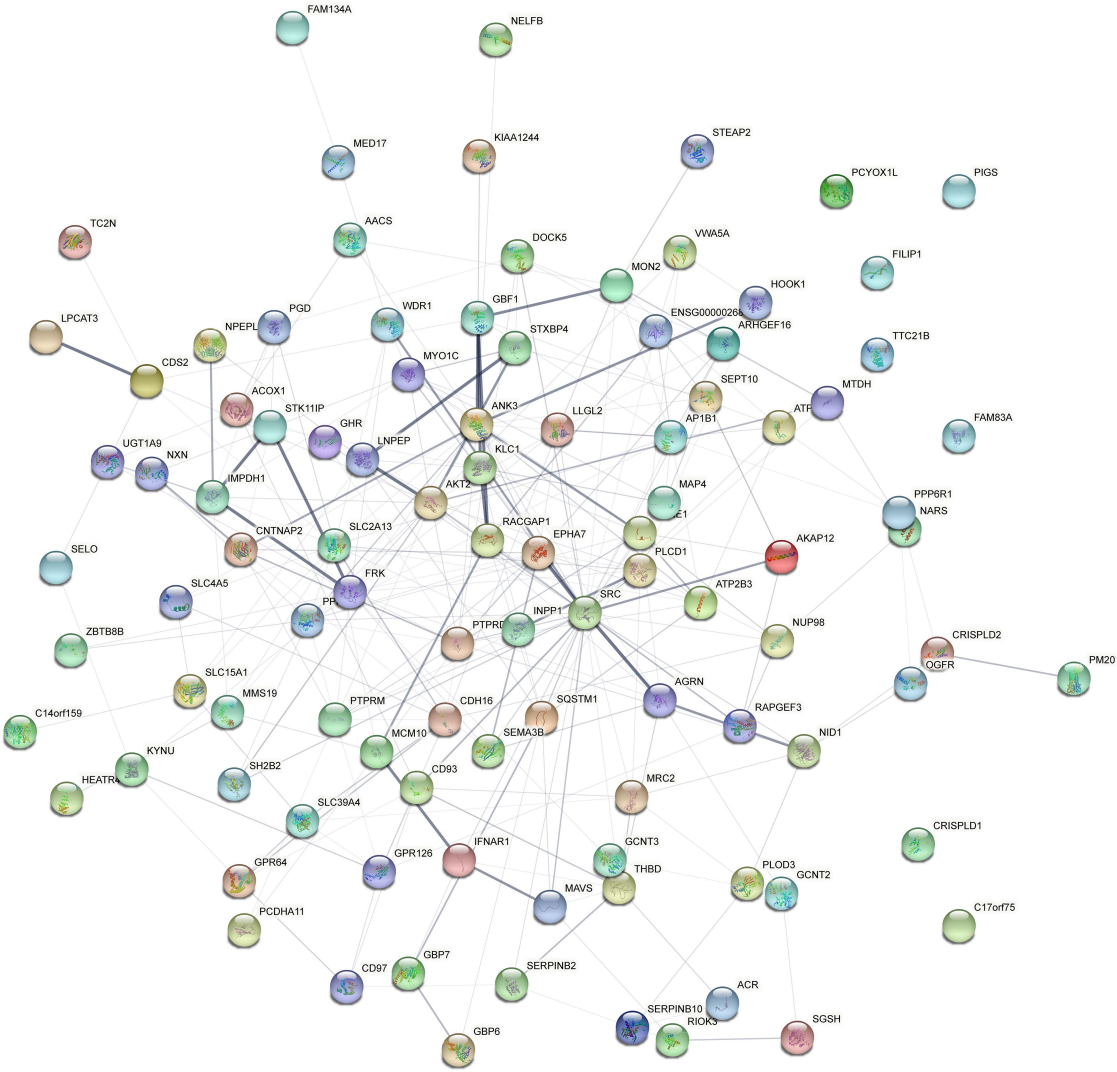
Protein–protein interaction (PPI) network analysis of the differential expressed proteins in the urine between VD and other two groups (AD and NC) using STRING. Each node represents a protein, and each edge represents a protein–protein association.

Four hundred eighty four overlapped proteins were identified by the comparison of VD with AD, and NC group, respectively, which could be considered as potential candidate biomarkers of VD (Figure 5A). Details of these DEPs were provided in Supplementary Table 4. The top 12 biological processes, the top 2 cellular components, and the top 6 molecular functions were similar to all the above except sorting differences (Figure 5B). In M pathways, 13 DEPs enriched in Carbohydrate metabolism, 8 DEPs enriched in Glycan biosynthesis and metabolism, 7 DEPs enriched in Amino acid metabolism, 6 DEPs enriched in Lipid metabolism. In Cellular Processes, 20 DEPs enriched in Transport and catabolism, 12 DEPs enriched in Cellular community – eukaryotes, 11 DEPs enriched in Cell growth and death. In Organismal Systems, 20 DEPs enriched in the Endocrine system, 19 DEPs enriched in the Immune system, 13 DEPs enriched in the Digestive system, 13 enriched in the Nervous system. In Human Diseases, 25 DEPs enriched in the Infectious disease: viral, 19 DEPs enriched in the Infectious disease: bacterial, 17 DEPs enriched in the Cancer: overview and 7 DEPs enriched in neurodegenerative diseases (Figure 5C). We used the STRING database that can generate a PPI network with tightly connected clusters shown in Figure 6. SRC-AGRN was the top one network node according to the combined score (Figure 6; Supplementary Table 5).

### 3.3. Candidate markers and composite biomarkers

**Figure 7:**
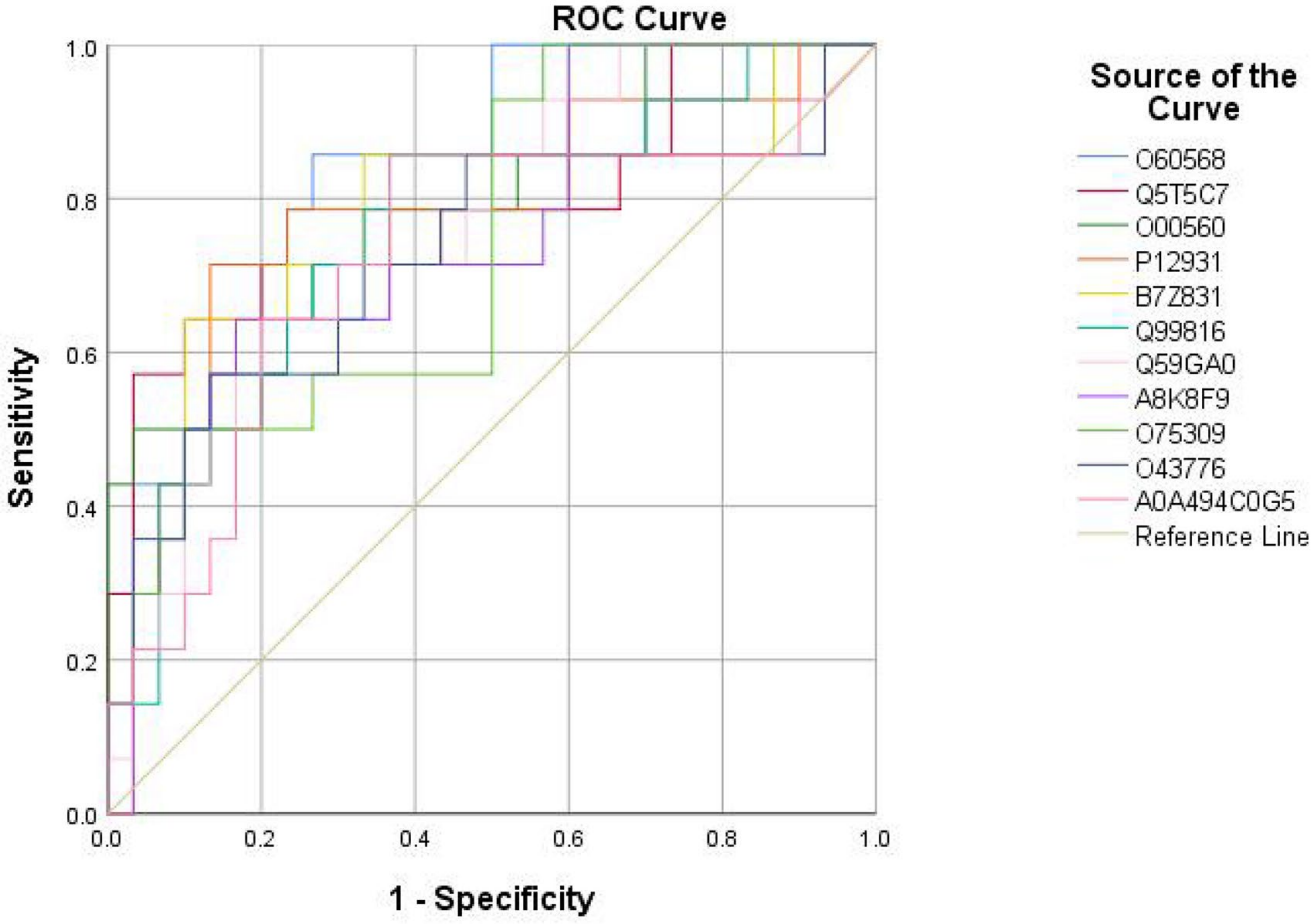
ROC curves obtained of the top 11 differential expressed proteins identified by proteomics analysis, including O60568 (PLOD3), Q5T5C7 (not named), O00560 (SDCBP), P12931 (SRC), B7Z831 (GPRC5B), Q99816 (TSG101, STP22, VPS23), Q59GA0 (THY1, CD90), A8K8F9 (PLCD), O75309 (CDH16), O43776 (NARS, asnS), A0A494C0G5 (AGRN).

Eighteen DEPs were selected as potential candidate markers from 484 common DEPs, which were expressed in all groups (more than 50% of the samples in each group were expressed as non-zero values) (Table 2). They were almost upregulated proteins and the diagnostic value of each single marker was evaluated by the receiver operating characteristic (ROC) curve analysis in the differentiation of the examined groups. The top 10 DEPs (named in KO (KEGG Ontology)) were statistically significant as candidate biomarkers to distinguish VD from AD or NC (shown in Table 3; Figure 7), including PLOD3, SDCBP, SRC, GPRC5B, TSG101/STP22/VPS23, THY1/CD90, PLCD, CDH16, NARS/asnS, AGRN.

**Table 2:** Urine differentially expressed proteins between VD, AD, and NC patients.

**Accession**	**Description**	**ko_id**	**ko_name**	**VD/AD**				**VD/NC**			
				**FC**	**Log2FC**	**P_value**	**Regulate**	**FC**	**Log2FC**	**P_value**	**Regulate**
O60568	Multifunctional procollagen lysine hydroxylase and glycosyltransferase LH3 OS=Homo sapiens OX = 9,606 GN=PLOD3 PE = 1 SV = 1	K13646	PLOD3	3.632	1.861	0.00397	up	2.032	1.023	0.004664	up
P12931	Proto-oncogene tyrosine-protein kinase Src OS=Homo sapiens OX = 9,606 GN=SRC PE = 1 SV = 3	K05704	SRC	3.612	1.853	0.007843	up	2.116	1.081	0.02818	up
A0A494C0G5	Agrin OS=Homo sapiens OX = 9,606 GN = AGRN PE = 1 SV = 1	K06254	AGRN	2.62	1.39	0.01611	up	1.792	0.8416	0.02939	up
A0A5C2GLK7	IG c58_heavy_IGHV3-23_IGHD5-18_IGHJ4 (Fragment) OS=Homo sapiens OX = 9,606 PE = 2 SV = 1			6.135	2.617	0.0169	up	2.757	1.463	0.006967	up
O43776	Asparagine--tRNA ligase, cytoplasmic OS=Homo sapiens OX = 9,606 GN=NARS1 PE = 1 SV = 1	K01893	NARS, asnS	2.224	1.153	0.01706	up	1.732	0.7924	0.02091	up
V9HW89	Epididymis secretory sperm binding protein Li 95n OS=Homo sapiens OX = 9,606 GN=HEL-S-95n PE = 2 SV = 1	K00008	SORD, gutB	6.035	2.593	0.01929	up	2.758	1.464	0.01519	up
Q99816	Tumor susceptibility gene 101 protein OS=Homo sapiens OX = 9,606 GN = TSG101 PE = 1 SV = 2	K12183	TSG101, STP22, VPS23	2.367	1.243	0.01963	up	1.731	0.7916	0.03069	up
Q5T5C7	Serine--tRNA ligase, cytoplasmic OS=Homo sapiens OX = 9,606 GN=SARS1 PE = 1 SV = 1	–	–	3.311	1.727	0.02475	up	2.864	1.518	0.002837	up
Q4JM47	AGR2 OS=Homo sapiens OX = 9,606 GN = AGR2 PE = 4 SV = 1	K20356	AGR2	6.053	2.598	0.02627	up	2.804	1.487	0.01157	up
A8K8F9	Phosphoinositide phospholipase C OS=Homo sapiens OX = 9,606 PE = 2 SV = 1	K05857	PLCD	2.377	1.249	0.02783	up	1.84	0.8797	0.03572	up
O00560	Syntenin-1 OS=Homo sapiens OX = 9,606 GN=SDCBP PE = 1 SV = 1	K17254	SDCBP	2.139	1.097	0.02855	up	2.29	1.195	0.000837	up
Q8N9U0	Tandem C2 domains nuclear protein OS=Homo sapiens OX = 9,606 GN = TC2N PE = 1 SV = 2	K17287	TC2N	3.455	1.789	0.03284	up	2.691	1.428	0.03307	up
B7Z831	cDNA FLJ55176, highly similar to G-protein coupled receptor family C group 5 member B OS=Homo sapiens OX = 9,606 PE = 2 SV = 1	K04619	GPRC5B	2.147	1.102	0.03795	up	2.018	1.013	0.02523	up
Q59GA0	Thy-1 cell surface antigen variant (Fragment) OS=Homo sapiens OX = 9,606 PE = 2 SV = 1	K06514	THY1, CD90	2.406	1.267	0.03981	up	2.406	1.267	0.03981	up
Q15046	Lysine--tRNA ligase OS=Homo sapiens OX = 9,606 GN=KARS1 PE = 1 SV = 3	K04567	KARS, lysS	4.635	2.213	0.04224	up	3.134	1.648	0.01185	up
B7ZM73	MON2 protein OS=Homo sapiens OX = 9,606 GN = MON2 PE = 2 SV = 1			4.622	2.209	0.04242	up	2.558	1.355	0.03145	up
O75309	Cadherin-16 OS=Homo sapiens OX = 9,606 GN=CDH16 PE = 2 SV = 1	K06810	CDH16	2.856	1.514	0.04312	up	2.134	1.094	0.02387	up
A6XND9	Beta-2-microglobulin OS=Homo sapiens OX = 9,606 PE = 2 SV = 1	–	–	2.285	1.192	0.04808	up	2.169	1.117	0.01165	up

**Table 3:** AUC statistics per protein for the classification status of VD group from AD and NC groups.

Test result variable(s)	Description	ko_id	ko_name	Area	Std. error^a^	Asymptotic Sig.^b^	Asymptotic 95% confidence interval	
							Lower bound	Upper bound
Q15046	Lysine--tRNA ligase OS=Homo sapiens OX = 9,606 GN=KARS1 PE = 1 SV = 3	K04567	KARS, lysS	0.602	0.111	0.279	0.385	0.820
**Q5T5C7**	Serine--tRNA ligase, cytoplasmic OS=Homo sapiens OX = 9,606 GN=SARS1 PE = 1 SV = 1	–	–	**0.786**	0.082	**0.002**	0.625	0.947
Q4JM47	AGR2 OS=Homo sapiens OX = 9,606 GN = AGR2 PE = 4 SV = 1	K20356	AGR2	0.645	0.107	0.124	0.435	0.856
V9HW89	Epididymis secretory sperm binding protein Li 95n OS=Homo sapiens OX = 9,606 GN=HEL-S-95n PE = 2 SV = 1	K00008	SORD, gutB	0.652	0.104	0.107	0.448	0.857
A0A5C2GLK7	IG c58_heavy_IGHV3-23_IGHD5-18_IGHJ4 (Fragment) OS=Homo sapiens OX = 9,606 PE = 2 SV = 1			0.655	0.103	0.101	0.453	0.857
Q8N9U0	Tandem C2 domains nuclear protein OS=Homo sapiens OX = 9,606 GN = TC2N PE = 1 SV = 2	K17287	TC2N	0.648	0.102	0.118	0.448	0.848
B7ZM73	MON2 protein OS=Homo sapiens OX = 9,606 GN = MON2 PE = 2 SV = 1			0.657	0.100	0.096	0.462	0.852
**O00560**	Syntenin-1 OS=Homo sapiens OX = 9,606 GN=SDCBP PE = 1 SV = 1	K17254	SDCBP	**0.783**	0.077	**0.003**	0.632	0.935
A6XND9	Beta-2-microglobulin OS=Homo sapiens OX = 9,606 PE = 2 SV = 1	–	–	0.545	0.105	0.632	0.339	0.751
O75309	Cadherin-16 OS=Homo sapiens OX = 9,606 GN=CDH16 PE = 2 SV = 1	K06810	CDH16	0.738	0.079	**0.012**	0.584	0.892
P12931	Proto-oncogene tyrosine-protein kinase Src OS=Homo sapiens OX = 9,606 GN=SRC PE = 1 SV = 3	K05704	SRC	0.776	0.082	**0.003**	0.616	0.936
**O60568**	Multifunctional procollagen lysine hydroxylase and glycosyltransferase LH3 OS=Homo sapiens OX = 9,606 GN=PLOD3 PE = 1 SV = 1	K13646	PLOD3	**0.831**	0.062	**0.000**	0.710	0.952
B7Z831	cDNA FLJ55176, highly similar to G-protein coupled receptor family C group 5 member B OS=Homo sapiens OX = 9,606 PE = 2 SV = 1	K04619	GPRC5B	0.769	0.085	**0.004**	0.603	0.935
Q59GA0	Thy-1 cell surface antigen variant (Fragment) OS=Homo sapiens OX = 9,606 PE = 2 SV = 1	K06514	THY1, CD90	0.757	0.075	**0.007**	0.609	0.905
A8K8F9	Phosphoinositide phospholipase C OS=Homo sapiens OX = 9,606 PE = 2 SV = 1	K05857	PLCD	0.755	0.078	**0.007**	0.601	0.908
A0A494C0G5	Agrin OS=Homo sapiens OX = 9,606 GN = AGRN PE = 1 SV = 1	K06254	AGRN	0.721	0.090	**0.019**	0.546	0.897
O43776	Asparagine--tRNA ligase, cytoplasmic OS=Homo sapiens OX = 9,606 GN=NARS1 PE = 1 SV = 1	K01893	NARS, asnS	0.726	0.091	**0.017**	0.548	0.905
Q99816	Tumor susceptibility gene 101 protein OS=Homo sapiens OX = 9,606 GN = TSG101 PE = 1 SV = 2	K12183	TSG101, STP22, VPS23	0.764	0.079	**0.005**	0.609	0.919

AUC, area under the curve; CI, 95% confidence interval.

The test result variable(s): Q15046, V9HW89, A0A5C2GLK7, Q8N9U0, B7ZM73, A6XND9, A0A494C0G5 has at least one tie between the positive actual state group and the negative actual state group. Statistics may be biased. a. Under the nonparametric assumption b. Null hypothesis: true area = 0.5.

The red represents *p* < 0.05, the bold represents the top 3 most prominent.

## 4. Discussion

VD and AD are the leading causes of dementia in the elderly, together accounting for more than 70 to 75% of cases (Jia et al., 2020). The etiology of AD comprise multiple genetic and environmental factors, which is characterized by amyloid plaques and neurofibrillary tangles, (Knopman et al., 2021) while VD is defined as loss of cognitive function due to hypoperfusion, ischemic or hemorrhagic encephalopathy, which attributable to cerebrovascular or cardiovascular pathologies (Iadecola, 2013). However, due to frequent overlap with AD and the lack of specificity and sensitivity of clinical diagnostic criteria currently, it is difficult to distinguish VD from AD in clinical practice (Karantzoulis and Galvin, 2011). Thus, it is important to explore ancillary biological markers as a complement to clinical assessments for the differential diagnosis.

In this study, the urine proteomic results showed quantitative changes in patients with VD compared to patients with NC and AD groups; among 4,699 identified urine proteins, 939 and 1,147 proteins displayed quantitative changes unique to VD vs. NC and AD, respectively, including 484 overlapped common DEPs. Then, 10 unique proteins named in KEGG database (including PLOD3, SDCBP, SRC, GPRC5B, TSG101/STP22/VPS23, THY1/CD90, PLCD, CDH16, NARS/asnS, AGRN) were confirmed by a ROC curve method. SRC-AGRN was the strongest network node according to the combined score according to the PPI network with tightly connected clusters generated by the STRING database (Figure 6). SRC-AGRN was the top one network node according to the combined score (Figure 6; Supplementary Table 5).

Firstly, Collagen is the major component of extracellular matrix. Collagen cross-link and deposition depend on lysyl hydroxylation.Lysyl hydroxylase is composed of lysyl hydroxylase 1(LH1), LH2a, LH2b and LH3, (Scietti et al., 2018) among which LH3 encoded by human PLOD3 has a variety of enzymatic activities and catalyzes lysine hydroxylation and subsequent glycosylation of collagen (Scietti et al., 2018) PLOD3 gene is localized on chromosome 7Q36 and plays an important role in the biosynthesis of collagen IV and IV (Valtavaara et al., 1998). Abnormal PLOD expression and loss-of-function mutations have been implicated in many collagen-related diseases such as bone fragility with arterial rupture and deafness, and contractures or LH3 deficiency (Salo et al., 2008; Vahidnezhad et al., 2019). Notably, some recent studies have demonstrated that LH3 is essential for the intermolecular cross-linking and stabilization of collagen IV. LH3 deficiency affects the assembly and secretion of collagen IV and vascular basement membrane (BM) integrity (Li et al., 2019). In addition, LH3 overexpression can reduce the susceptibility of hypertensive ICH, and its regulation may be a feasible method for the prevention of intracranial hemorrhage (ICH) in the future (Li et al., 2019). In our study, the high expression of LH3 in VD may indicate an enhanced vasoprotective effect, which is distinct from AD and NC.

Another important protein marker is SRC, a member of the SRC family of non-receptor protein tyrosine kinases (PTKs), many of which are essential for growth, proliferation, and differentiation (Kalia et al., 2004). Five Src family kinases (SFKs) are expressed throughout the CNS in humans (including Src, Fyn, Yes, Lck and Lyn) and participate in many cellular functions (Ali and Salter, 2001). Specifically Src, as a key regulator of the NMDA (N-methyl-D-aspartate) subtype of glutamate receptors (NMDARs) and NMDAR-dependent synaptic plasticity and excitotoxicity may be crucial for learning and memory, pain, epilepsy and neurodegeneration (Yu and Salter, 1999; Salter and Kalia, 2004). Excitotoxicity mediated by NMDAR is associated with neuronal death in many pathological conditions, including CNS trauma, ischemia and neurodegeneration. This suggests that Src-mediated pathways and tyrosine phosphorylation of NMDAR may contribute to the pathophysiology of ischemic neuronal death in VD patients (Takagi et al., 1997, 1999; Cheung et al., 2003).

As shown in our result, AGRN-SRC was the top point of convergence according to the PPI network. Agrin (AGRN) is a kind of heparan sulfate proteoglycan rich in cerebrovascular basement membrane. It is a secreted neuroprotein, which is essential for synapse formation and stability (Bogdanik and Burgess, 2011). It is expressed in different tissues and non-neuronal cells, such as brain, lung, heart, kidney etc., and has been found to be regulated in various disease conditions, such as diabetes, cardiovascular, pulmonary, renal, muscle wasting, nerve and brain damage, and neurodegenerative diseases, and subsequently explored as a biomarker (Donahue et al., 1999; Rauch et al., 2011; Drey et al., 2015; Rauch et al., 2018). It has been reported that AGRN is important for maintaining the composition of the blood–brain barrier (BBB) in mouse models of AD and that changes in AGRN expression (especially vascular-associated AGRIN) affect a-amyloid (AA) homeostasis (Barber and Lieth, 1997; Berzin et al., 2000). Similarly, the effects of altered Agrn expression in the brain of VD patients affecting cerebrovascular and BBB may also be part of its pathological mechanism. However, how SRC-AGRIN interaction act in VD remains to be investigated. Perhaps advances in genetics and proteomics will provide answers to unanswered questions in this exciting field in the future.

In addition, we found a very interesting question: why are the majority of dysregulated proteins in VD upregulated proteins?

Nevertheless, this study also has certain limitations. First, the samples included were too limited to adjust the effects of some other factors, such as age, sex, blood pressure levels, potential drug effects etc. Second, VD is often difficult to distinguish from AD and mixed dementia because of the limited sensitivity and specificity of clinical criteria. Second, in urine proteomic studies, proteomic variability cannot be avoided, as has been demonstrated in many previous studies, (Nagaraj and Mann, 2011) due to technical variation or many physiological factors such as daily, gender, aging, diet, exercise, and intra - and inter-individual variation. Third, it is also a limitation that there is no cross-verification using any other biological specimen/analytical technique. Therefore, given the limitations of the research, our team is currently collecting more samples of patients and is preparing to further verify the specificity of each possible differently expressed protein using targeted proteomics and ELISA/Western Blot technique. We will expand the sample size, increase stratified analysis to adjust complicated influencing factors, standardize sampling procedures and develop more stringent *p*-values to attenuate this variability by continuing in future studies. In addition, we will also conduct in-depth analyses of VD proteomes and post-translational modifications (PTMs) to identify high-confidence biomarker candidates for VD by integrating multiple proteomes including cortex, cerebrospinal fluid, and serum in the future, as in previous AD studies (Wang et al., 2020).

## 5. Conclusion

This study demonstrated that we successfully screened ten upregulated urinary protein biomarkers of VD different from AD and NC using HPLC-MS/MS analysis, which is an effective and specific method. In addition, VD and AD share some urinary proteins, suggesting that they may share some similar pathophysiological mechanisms. SRC-AGRN interaction may act an essential role in VD pathological mechanism, which merit further attention.

## Data availability statement

The datasets presented in this study can be found in online repositories. The names of the repository/repositories and accession number(s) can be found at: mass spectrometry proteomics data have been deposited to the ProteomeXchange Consortium via the PRIDE partner repository with the dataset identifier PXD022189.

## Ethics statement

The studies involving human participants were reviewed and approved by this study was approved by the Ethics Committee of the Second Xiangya Hospital of Central South University, and all participants provided written informed consent following the Declaration of Helsinki and the independent ethics committee or institutional review board. The patients/participants provided their written informed consent to participate in this study.

## Author contributions

YZ designed the research and determined the structure of the paper. RC selected the references and contributed to the writing. YY collected the clinical data. WX and BZ helped to analyze experiment results. LZ contributed to the revision and finalization of the article. All authors contributed to the article and approved the submitted version.

## Funding

This study was supported by the National Science and Technology Fundamental Resources Investigation Program of China (2018FY100900), the National Natural Science Foundation of China (81571151 and 81641039), and the Hainan Provincial Natural Science Foundation of China (822QN499).

## Conflict of interest

The authors declare that the research was conducted in the absence of any commercial or financial relationships that could be construed as a potential conflict of interest.

## Publisher’s note

All claims expressed in this article are solely those of the authors and do not necessarily represent those of their affiliated organizations, or those of the publisher, the editors and the reviewers. Any product that may be evaluated in this article, or claim that may be made by its manufacturer, is not guaranteed or endorsed by the publisher.
